# Working in biocuration: contemporary experiences and perspectives

**DOI:** 10.1093/database/baaf003

**Published:** 2025-02-12

**Authors:** Sarah R Davies

**Affiliations:** Department of Science and Technology Studies, University of Vienna, Universitätsstraße 7, 6. Stock (NIG), Vienna, 1010, Austria

## Abstract

This perspective article synthesizes current knowledge regarding what is known regarding biocuration as a career and the challenges facing the field. It draws on existing literature and ongoing qualitative research to discuss the nature of biocuration, biocurators’ career trajectories, key challenges that biocurators face, and strategies for overcoming these. Overall, biocurators express a high degree of satisfaction with their work and see it as central to the wider biosciences. The central challenges that they face relate to the underfunding and under-recognition of this work, meaning that there is minimal stable funding for the field and that the work of human biocurators is often invisible to those who use curated resources. The article closes by critically discussing existing and potential strategies for responding to these challenges.

## Introduction

There is a long history of data curation within the biosciences. As Bruno Strasser has shown in his book *Collecting Experiments: Making Big Data Biology*, biology has always involved ‘collecting, comparing, and classifying objects in nature’ [1], whether in databases or in earlier formats such as museums, libraries, or natural history collections [2]. Biocuration as a recognized profession has, however, emerged more recently. The International Society for Biocuration (ISB) was formed in 2009, partly with the aim of consolidating and raising the profile of the field [3], while this journal was similarly launched in 2009 to solve the problem that ‘the description of a database and the tools to interact with it are not deemed essential to the communication [in other venues]’ [4]. There have been calls since at least 2008 to increase ‘structure, recognition and support’ for biocuration, a field that ‘links biologists and their data’ [5] and that relies on the efforts of biocurators, a community that has been called the ‘unsung heroes of molecular biology’ [3]. Accordingly, the ISB carries out a range of activities that aim both to support best practices in biocuration and increase its visibility to research funders and database users [6].

There have been a number of efforts to research the experiences of those working in biocuration since its emergence as a field of professional practice. It is clear, for instance, that a majority of biocurators identify as women, have PhDs, and experience high levels of satisfaction in their work [7–10], as well as that in practice biocurators may have a wide variety of job titles and rarely hold permanent positions [7, 11]. In this perspective article, I add to these discussions in two ways. First, I draw together this literature to synthesize what is known regarding biocuration as a career and the experiences of those working in it. Second, I combine this with findings from ongoing qualitative research into the sociology of biocuration (see [12]), using this material to reflect on practical implications for biocuration and biocurators. The article thus serves as a summary of biocurators’ experiences of working in the field and offers perspectives and proposals regarding the issues that they face.

In what follows, I document current knowledge in a number of areas. First, I discuss the nature of biocuration; second describe what is known about biocurators’ career trajectories; third summarize key challenges that curators face; and, finally, critically reflect on strategies for overcoming these. Findings from these sections are summarized in Table 1. Before moving on to discuss these findings in more detail, however, I begin by briefly introducing my research and the materials on which I am drawing.

**Table 1.** Summary of findings structured around the four questions that the article explores

What is biocuration?	Biocuration is data work that adds value to and increases the accessibility of bioscience data. It is understood as collaborative, expert work, and as being oriented to service and to carefulness.
What do careers in biocuration look like?	Biocurators’ career experiences differ, but individuals tend to express high satisfaction with the work of biocuration. They often enter it ‘by accident’ and experience challenges with career progression.
What challenges do biocurators face?	The difficulty of securing long-term, stable funding for biocuration and biocurators is fundamental. Other challenges—such as the precarity of biocuration work, its lack of visibility and status within the wider biosciences, and the under-curation of data—are related to this lack of support.
What strategies can be used to overcome these challenges?	Existing strategies include professionalizing, increasing visibility of biocuration, and finding means of crediting biocuration activities via authorships and in researcher identifiers. More speculative strategies involve emphasizing the epistemic contributions made to the biosciences by biocuration, and exploring parallels and potential learning from other forms of data work.

## Materials and methods

This article is, as noted, somewhat unusual in that I both synthesize existing literature and present empirical findings. I am working in a tradition of ethnographic research that involves participant observation in a particular culture (here, the professional community oriented to biocuration) and that therefore draws on a diverse range of materials that offer insights into that culture [13]. As part of my interest in the sociology of biocuration—the ways in which working as a biocurator is ‘made sense of’ [14]—I follow and review the scholarly literature on the topic, both from the field itself (e.g. [15]) and from social science studies of it (e.g. [16]). I conduct both digital and in-person ethnography by engaging with websites, on- and off-line events, and following discussions on mailing lists or social media [17, 18].

In addition to this ethnographic research, my discussion here draws on 15 qualitative interviews carried out in 2022 by myself and my colleague Constantin Holmer with individuals working across the field of biocuration [12]. These interviews were semistructured [19], meaning that we moved flexibly through a list of topics that included interviewees’ career trajectories, experiences of biocuration, and descriptions of what curation work involved in practice (this topic list is included as Supplementary Material; the semistructured nature of the interviews meant that the planned topics were covered in each interview, but that the order varied depending on whether interviewees themselves raised particular topics). The interviews took place online via Zoom or another communication platform and were recorded, transcribed, and pseudonymized in order to protect the confidentiality of those we spoke to. Recruitment was carried out using suggestions of key actors within biocuration from the ISB and ‘snowball sampling’, whereby interviewees suggested other biocurators we might speak to. Interviewees were based in Europe, North America, and Africa; 10 worked in academic institutions, while 5 were based in bioscience companies closely connected to academic research. Throughout the article, I include quotes from the interviews to illustrate key themes or patterns but do not give any information about interviewees that could be used to identify them (such as national location or the resource on which they work).

The following article makes use of all these different forms of material to synthesize an account of contemporary experiences of careers in biocuration, seeking to describe, in the manner of much ethnographically oriented qualitative research, central patterns and taken-for-granted knowledge within the culture under study [20] The aim, as Van Maanen writes, is to capture (some of) ‘the language, concepts, categories, practices, rules, beliefs, and so forth, used by members of the written-about group. These are the stuff of culture’ [20]. I therefore use illustrative quotes from the interviews to add texture and nuance to the overall findings. When writing for participants in a particular culture—in this case, those working in or around biocuration—the goal is in many ways to present what is experienced as obvious or even banal; my hope, then, is that this synthesis resonates with readers.

## What is biocuration?

The published literature contains a number of definitions of biocuration. Most prominently, the ISB describes biocuration as involving ‘the translation and integration of information relevant to biology into a database or resource that enables integration of the scientific literature as well as large data sets’ [6]. Others focus on what is achieved through biocuration—for instance, by noting that the ‘primary role of professional biocurators is to extract knowledge from biological data and convert it into a structured, computable form via manual, semi-automated and automated methods’ [10] or that its aim is ‘to extract valuable knowledge from the corpus of available biological data and to accurately represent it in a computable manner so that it may be easy to understand and disseminate’ [21]. Biocuration thus involves ‘adding value’ to biological datasets [11, 22], turning information into knowledge, or allowing it to be accessed in easier or new (and particularly computable) ways. As such, it is central to contemporary biosciences in that it allows better use of the vast quantities of biological data that are now being produced (a ‘data deluge’ [23]). On the one hand, it organizes and renders this accessible via databases, while, on the other, biocuration ensures that these data are structured so as to allow for new insights and knowledge by enabling ‘complex querying and advanced exploitation of distributed data’ ([24], cf. [25, 26]). Databases are a central product of biocurators’ work, but curators may also work on knowledgebases or other types of research infrastructure. In this article, I use the term ‘resources’ to capture all such products [27].

What does this mean in practice? Both literature and interviews with biocurators indicate that biocuration involves a range of overlapping activities including, but not limited to, reading scientific literature in a particular domain area and extracting data and/or information from this, integrating datasets into databases or other resources, annotating data by applying bio-ontologies or adding meta-data, developing or extending such bio-ontologies, and, importantly, liaising with user communities and with others working in biocuration [21]. While there are a number of core skills biocurators are expected to have—in particular, specialist knowledge of a particular biological domain, experience of working with data and metadata (including data storage and lifecycles), and technical skills (such as programming in particular languages or experience with relational databases [6, 28])—in practice, biocurators may focus on different aspects of the role and have jobs where their responsibilities also cover related activities such as ontology engineering, bioinformatics research, software or platform development, community outreach and engagement, or supporting the creation and sharing of Findable, Accessible, Interoperable, and Reusable data [8].

Two features are particularly important with regard to the day-to-day tasks that biocuration involves. The first is that much of this work is ‘expert’ work, requiring high-level knowledge of the domain that is being curated. While there have been efforts to encourage community curation (where curation is carried out, or at least begun, by the scientists producing the data [29]) and to train and involve students in biocuration [30, 31], the points in biocuration pipelines that require human curation exactly tend to be those that require expertise in interpreting and assessing published findings in a specific field. As one interviewee told us,

the core of the job is interpreting the scientific literature, understanding what the researcher was trying to get at and then classifying that information in a very formal and structured way … a lot of the work time we spend, we read papers, try to understand the science.

It is thus not coincidental that a majority of biocurators have higher degrees (such as PhDs) and, frequently, postdoctoral experience working in related areas of bench science [7]: often, having such experience is central to being able to curate particular kinds of data.

A second point that is worth noting is that many of the skills that biocurators frame as important to their work are ‘social and interactional skills’. The ISB’s ‘Biocuration Generic Job Description’, for instance, highlights both ‘Ability to collaborate and work in a team’ and ‘Can communicate well with computer programmers, bioinformaticians and biologists alike’ as typical job requirements [6], while interviewees similarly talked about the collaborative and coordination aspects of their roles. Biocurators’ work can include engaging with user communities, raising funding, acting as intermediaries between scientists and software developers, discussing curation decisions with other curators on a team, and—particularly important in the case of ontology development and maintenance—coordinating and implementing standardized practices across many global sites. While biocuration may involve periods of intense focus and solitude, it is thus framed as collective and collaborative in a way that other areas of science may not be. This sense of collectivity is heightened by a widely shared commitment to biocuration as securing a public good: the creation of resources, many people told us, was a ‘public service’.

As well as biocuration involving a particular set of activities, biocurators also talk about the kind of work that it is—that is to say, biocuration is represented as having a particular style or ethos, or as benefiting from particular characteristics. This can be summarized as relating to service, on the one hand, and precision and carefulness, on the other [16, 32]. As already noted, biocuration is integral to contemporary biosciences and plays a central role in knowledge sharing and the creation of new insights. As St. Pierre and McQuilton ask, rhetorically, ‘If a discovery is made and no one knows about it, is it really a discovery?’ [9]. Biocuration is thus framed as a vital service that allows contemporary bioscience research to take place as efficiently as possible and that enables fuller utilization of the vast quantities of data that are now available. While some have pointed out that this emphasis on service runs the risk of becoming ‘an obstacle to [curation’s] recognition as an important part of science in its own right’ [16], it is also one aspect of what makes biocuration rewarding to many curators:

… the users of the database, they seek us out and they come up to us, and they thank us […] and say, oh my gosh, I could have never done my PhD thesis without your help, or I got this really big grant because of the data in the database. So we get to actually interact with the people who use the data, and that is very definitive, like they clearly were able to design a project or get a PhD because of what we did. So that’s very rewarding.

As well as the field being oriented to service and to ‘helping’ [12], biocurators speak about their work as involving precision, attention to detail, and being meticulous—always ‘dotting the i’s and crossing the t’s’, as one interviewee said. This orientation is again well illustrated by the ISB’s generic job description, which begins with the lines ‘Have you ever been called pedantic or precise? Did you take that as a compliment, not a criticism? If so, we have the job for you!’, and continues by noting that ‘The best biocurators are detail oriented, conscientious, and good communicators. They are adaptable to the needs of the community and/or to the needs of the software systems’ [6]. As Ane Møller Gabrielsen has argued, biocuration is ‘careful’ work—‘life science data care’ [32]—that involves caring for science (and a user community) through attention to detail, precision, and accuracy. Again, this is something that curators find satisfying, both in exploiting their ‘quirky traits’ and ‘perfectionism’ and in using these to create a ‘public service’:

I suppose the motivation is belief in what you’re doing. And you have to believe that what you’re doing is useful. Believe that you’re helping people. Caring that you do a professional job but you do it well enough that you can hand on heart put something out there in the public domain and know that it’s the best that you can do.

In sum, in answering the question ‘What is biocuration’, we can say that it is a form of data work that adds value to and increases the accessibility of bioscience data, that it is collaborative expert work, and that it is oriented to service and to carefulness.

## What do careers in biocuration look like?

Perhaps unsurprisingly given the relative novelty of the field as a specific professional domain, there is no single standard trajectory into biocuration or well-established career pathway through it. Those we spoke to had different experiences, had come into biocuration through different routes, and were working in a variety of types of positions. There were, however, some commonalities: many people told us that they had ‘fallen into’ the field or entered it through a combination of happenstance (while thinking about what to do next, for instance, ‘all of a sudden I had an email [about a biocuration job] from the mailing list’) and practical drivers (such as needing to work remotely or flexibly). Several people spoke about wanting to move away from bench science—many biocurators, one interviewee told us, ‘weren’t keen on lab work but loved reading about science’. Others were drawn to the possibility of working with the state of the art of knowledge in a whole field rather than a tiny sub-section of it—as one curator said, contrasting their experiences of a PhD in a wet lab to their current work:

it’s nice to have a bit more of an overview perspective. So not just have this small problem that you’re trying to solve but have a more global view of things and see things in a big picture. I really enjoy that.

While the curators we have spoken to express a high degree of satisfaction with and pleasure in their biocuration work, they generally only discovered this once they had ‘fallen into’ curation rather than this being a possibility that they had known about from early on in their biosciences careers. For others, their biocuration work was essentially a passion project that they managed to fit around other types of scientific work—something that was rendered necessary by the challenges of funding biocuration projects—with several people describing funding their curation activities ‘through the back door’ through developing more traditional research projects.

This heterogeneity within biocuration careers is supported by other sources. It is clear, for example, that biocurators hold a wide range of job titles, even when working in the same institution [10, 11], a feature that adversely affects career progression because ‘a lack of standardized names and titles’ means that ‘there is not a widespread understanding of what a biocurator does and what a typical career progression should look like’ [10]. Survey results show both high satisfaction and a degree of longevity in working in the field (in 2021, 62% of respondents had been working in biocuration for >10 years [10]) but also limited career progression: the same survey noted that ‘Less than half of respondents (49.6%) said they have been promoted during their career’ [10]. Similarly, the majority of those working in biocuration are on temporary contracts or are dependent on raising funding for their positions [7]. These issues have been a focus for the ISB in recent years, with efforts such as career workshops and the provision of resources (such as the generic job description quoted above) that aim to support biocurators’ career development and progress. However, it seems likely that many of these challenges need to be resolved at the level of institutions or funders. As Holinski *et al*. suggest, ‘we perhaps need a new model of careers in biocuration … we recommend that both academia and industry provide clearer career structures for scientists [for] whom biocuration is their primary activity’ [11].

Aside from these challenges (which I will discuss further in the next section), there are two issues worth noting with regard to career pathways in biocuration. The first is the pattern of biocurators ‘falling into’ the field—finding it more or less accidentally and discovering that they loved it (cf. [11]). As one curator told us:

biocuration never came up as an opportunity, and I never met anyone that worked in biocuration. […] I just ended up doing a little bit of teaching, and I was looking for jobs. And this was just a job ad I came across, and had no idea what it was. But I applied for the job […] even when I started, I was like, I’m not sure what I’m supposed to be doing here. And then it all kind of came together, and I was like, this job is made for me. I love biocuration.

The issue here is biocuration’s lack of visibility in the wider biosciences, a status that connects to other challenges (discussed below) but that also has the concrete effect that researchers (at any career stage) are probably not aware of it as a possible pathway. It is likely that many others who might also ‘love biocuration’ and find it a good fit for their skills are not being recruited because of this.

Second, it is also clear that there are very few formal educational programmes in biocuration. While there are an increasing number of resources to support training in biocuration—with the European Bioinformatics Institute of the European Molecular Biology Laboratory (EMBL-EBI’s) collection of online courses being one example [33]—there are currently no degree programmes or postgraduate certificate courses in the field. While biocurators do report an interest in training opportunities of different kinds [11], the importance of increasing such opportunities may be symbolic as well as practical. Scholarship in the sociology of professions, which has studied the ways in which particular forms of work become ‘professionalized’ as coherent (and visible) fields of practice, suggests that educational programmes and accreditation are important in solidifying an area of work as a distinct, recognizable area of work [34]. While developing such programmes is not straightforward—exactly because they involve codifying, and therefore deciding, what skills and content are central to a particular profession, and because in practice many skills can and perhaps should be learned ‘on the job’—it is therefore worth considering the ways in which forms of accreditation may help to ‘achieve recognition for a legitimate career’, as one interviewee told us.

In sum, in answering the question ‘What do careers in biocuration look like?’ we can say that, while biocurators’ experiences differ, individuals tend to express high satisfaction with the work of biocuration but enter it ‘by accident’ and experience challenges with career progression.

## What challenges do biocurators face?

In this section I consider in more detail challenges and frustrations that biocurators report. Several of these have already been suggested; here, however, I document more fully a set of linked issues that are experienced as threatening the careers and well-being both of individual curators and the field as a whole.

It will come as no surprise to readers that the issue of funding for databases (and other resources) is a central concern for biocurators. In almost all cases, funding for the creation and maintenance of resources—including those that are extremely widely used—is fundamentally precarious, requiring, at best, constant efforts to patch together funding for infrastructure and the curators who work on it, or, at worst, resources being abandoned or maintained only through volunteer labour. Indeed, it is clear that every few years new crises around key resources emerge and come to public prominence, with—thus far—few long-term solutions being put into place [35–37]. Biocurators thus consistently talked about their work being shaped by funding dynamics, as in the two quotes below:

To this day, despite the importance of curation, funders in Europe and the United States have a problem understanding the importance of curation.

… funding has always been an issue, and it’s something that’s discussed at every single biocuration conference; how do we justify the funding? How we make sure there is funding for it? […] the people doing the experiments, they’ve got funding to do the experiments, and that’s it. And then someone else has to come up with the money to put their experimental data into a searchable format.

The second quote, in particular, suggests some of the reasons that securing funding for biocuration is challenging. Funders largely fund research projects, which are expected to take a particular form—carrying out experiments, in the example given above. With some exceptions (for instance, for certain types of large-scale research infrastructure or equipment), funding focuses on the production of novel results and on track record as measured by, in particular, high-profile research publications. Activities such as curation, the maintenance of resources, or ontology development do not fit into the logics that research funders use to assess quality and allocate funds—hence, as we have already seen, biocurators funding their work ‘through the back door’ via other kinds of funded research, or carrying it out in their spare time. This lack of funding for the field as a whole is experienced as having impacts both on the quantity and speed of curation that is possible—‘instead of having seven people do five papers a week, you could have two people do five a week’, one curator said. ‘And then we just won’t be able to keep up with the literature’—and its quality, and thereby the extent to which datasets can be utilized by researchers: ‘if we want to do wide-ranging research, then we have to have data that are properly annotated’, said another.

I have been unable to find any data or publications examining the relative rates or likelihood of funding for biocuration projects compared to other areas of science. Scientometrics research has tended to focus on the impacts of research funding (for instance on researchers’ productivity [38]) rather than its likelihood, and has noted the challenges of studying funding dynamics, given their complexity [39]. More generally, based on qualitative research Grit Laudel has argued that ‘a funding proposal’s success depends on several factors that are not linked to quality and cannot even be controlled by scientists’ [40], while Laudel and Gläser suggest that research proposals involving ‘planned innovations’ are more likely to be funded [41]. In the context of biocuration, it therefore seems likely that a (perceived) lack of novelty or innovation in biocuration activities hinders the likelihood of funding.

Funding of resources and of their own time is thus a central issue for biocurators. A related challenge is the human impacts of this precarity. We have already seen that relatively few curators have permanent positions, even at senior levels. Biocurators often talked about the stress involved in the fact that they are not only seeking to secure the continuity of the resource they work on, but their own positions:

… if I wrote four grants, I’d get one. And it’s very time-consuming writing grants and it’s very emotional. I think, one day I got rejected from two grants on the same day that had taken me, you know, three months for each grant to write […] having been told that I would only have a job if I had funding, you know, it makes it even more emotionally challenging when you get somebody saying, yeah, we decided that wasn’t good enough. Some of the rejections have been awful.

Practically and emotionally dealing with the precarity of their work was thus a central challenge for biocurators. Similarly, biocuration’s lack of visibility and, in some contexts, status was an issue that frustrated many (already demonstrated in the interview quote above where funders are framed as having ‘a problem understanding the importance of curation’). The fact that users of curated resources often continue to be unaware (or do not acknowledge) that expert human labour is involved in creating these was a source of humour but also of frustration. When attending conferences, one biocurator said, delegates told them how much they loved the resource but were ‘kind of shocked that there’s humans there’. (A discussion of the emerging use of AI in biocuration is outside the scope of this article, but it is worth noting that interviewees agreed that automated curation methods would be a ‘support’ to human curators but never fully replace them. High quality curation was seen as needing a ‘human brain’.) Just as had been the case for many curators before they entered the field professionally, there was minimal knowledge from users about how resources were created (and funded) and very limited awareness of biocuration as a professional space. This invisibility had clear implications for funding—funders who would say, ‘well, why don’t you do this with AI?’, as one interviewee told us—but also with regard to understanding biocuration as requiring a high degree of domain knowledge and other forms of expertise:

I think my [user] community think of me as a scientist, but I think that’s taken time, and I think a lot of communities don’t think of their database providers as true scientists. They think of them more as computer people […] there are definitely bench scientists who don’t think that biocuration is a science.

Again, this view of biocuration—which was based, this interviewee said, on a misunderstanding of the kinds of complex analytical work involved in biocuration—shapes both funding possibilities (as with an interviewee who had been told by a funder that what they were doing was ‘not research’ and therefore not fundable) and its status in the wider biosciences. Biocurators, therefore, had to fight against a view of their work—whether implicitly or explicitly expressed—that downgraded its significance and complexity [32] and that implied, as Bruno Strasser has written concerning funder reactions to early database curators, that biocuration is ‘simply clerical’ in nature [1] (Strasser’s [1] account, which explores the history of approaches to knowledge production in the biological sciences, tells the story of several early databases and the curators who developed them. His discussion of the creation of the *Atlas of Protein Sequence and Structure* in the 1960s, and in particular the work of Margaret O. Dayhoff, makes it clear that many of the challenges that biocurators currently face were present even in these early days: projects such as the *Atlas* ‘simply did not fit within the standard categories of science funding’ [1] and were therefore constantly under financial pressure.)

Other challenges are related to the work itself. Exploring the culture of biocuration reveals a picture of the vast mass of data (and related literature) that is emerging from the biosciences, the gathering momentum of this, and biocurators, as a small but dedicated community, desperately seeking to manage and organize this ever-increasing mass. It is clear that there is far more work to be done than is possible under current conditions: ‘the volume of work we have to get through’, said one curator, ‘is way more than, it’s probably ten times the amount at least, than there are funded biocurators on the planet’. On the one hand, this offered biocurators choice: as one interviewee said, ‘because there’s so much underfunding, you can pick whatever you want to do because nothing is over-curated’. On the other hand, however, this meant that much of the data being generated goes uncurated or is invisible to the wider communities who might make use of it, thus losing the additional value that might be added to it through curation. A related issue was, for at least some curators, the challenges of working with literature or datasets that made curation difficult. Biocurators have much to say regarding the inconsistencies and redundancies that are present within scientific literature, and the inefficiencies that these produce:

we deal with data sets. They’re very messy. You know, they’re not structured. We’re reading the literature; it’s a complete mess. Every author says the same thing a hundred different ways. And so by finding those ontologies that formalize the terminology that we need, we can translate what the author said to its actual proper name.

Curating data thus not only involves identifying and understanding relevant literature but also standardizing the information contained within it. Indeed, one central challenge continues to be consistency and the avoidance of redundancy and replication, for instance in the development and use of bio-ontologies [42].

In sum, in answering the question ‘What challenges do biocurators face?’ we can say that the difficulty of securing long-term, stable funding for biocuration and biocurators is experienced as fundamental. Other issues—such as the precarity of biocuration work, its lack of visibility and status within the wider biosciences, and the under-curation of data—are related to this lack of financial support for biocuration.

## What strategies can be used to overcome these challenges?

While working in biocuration is both experienced as personally rewarding and satisfying, and while it is central to the utilization of the vast quantities of data being produced in the biosciences, biocurators, therefore, face a range of challenges in carrying out their work. In this section, I discuss existing and potential strategies for overcoming or responding to these (also summarized in Table 2).

**Table 2.** Summary of current and potential strategies for overcoming the challenges facing biocuration

Central strategy	Examples
*In existence or in progress*	
Increase the visibility of biocuration	Outreach to user communities Highlight the work of biocurators on resource interfaces Engage funders
Professionalize biocuration	Codify job expectations Develop educational programmes and accreditations in biocuration
Find ways of crediting biocuration	Allow authorships based on curation contributions Make curation visible in ORCIDs
*Speculative*	
Emphasize biocuration’s epistemic contributions	Reframe biocuration as shaping contemporary bioscience research
Explore parallels and learning from other forms of data work	Use concepts from other domains in arguing for biocuration’s status and importance Learn from strategies developed by other data workers

One approach has been to seek to overcome biocuration’s invisibility to its users and funders. Biocurators already carry out a range of outreach activities to their user communities (such as attending conferences) that raise the profile of resources and the teams behind them, while some that we have spoken to have talked about trying to make human curation more visible and prominent on resource interfaces so that it is more difficult, as a user, to ignore the expert human work that is involved in them. The ISB is central to these awareness-raising efforts. An important part of its mission is to help ‘define the profession of biocuration’ [6] and, more generally, to promote biocuration throughout research and funding systems. Many of its activities are oriented both to increasing the visibility of biocuration generally and supporting biocurators as they develop their careers. Resources such as the ‘Biocuration Generic Job Description’ and the collection of training opportunities on the ISB website are central efforts towards solidifying biocuration as a distinctive research community with specific skills and professional expertise. In the same way, formal educational programmes that result in certification or accreditation in biocuration from widely recognized institutions are something that many biocurators see as being important. The University of Cambridge’s Postgraduate Certificate in Biocuration (which ran in 2020–21 but apparently not afterwards [43]) is one example of what such a programme might look like—though recruitment depends on potential participants being aware enough of biocuration to be interested in studying it further.

Related efforts aim to render biocuration more visible to and legible within funding and reward structures within research systems. Journals such as this one enable the publication of descriptions of databases, allowing curators to disseminate their work in article form—a type of output that is generally valued in academic recruitment or evaluation and that will therefore help curators with career progression in academic settings. Similarly, the possibility of attaching DOIs to datasets upon their deposition (connecting these to ORCIDs) renders it easier to gain credit and visibility for the production and preparation of shareable data. Discussions around the nature of authorship and efforts to expand the types of contributions that can be credited and made visible in publications are also relevant here. Contributor Role Ontologies and Taxonomies, such as the Contributor Roles Taxonomy, allow diverse kinds of work to be credited in publications, including data curation [44, 45]. Taking a slightly different approach, APICURON ‘gamifies’ curation contributions (including community curation) to track and make visible biocuration at both individual and resource levels [46]. While APICURON integrates its own reward mechanisms—such as medals and a leader board—it can also be integrated with ORCID, so that contributions to particular resources are made visible on an ORCID profile. At a structural level, efforts such as the Global Biodata Coalition are attempting to engage research funders with the need to stabilize funding for at least core data resources, including by mapping the resource landscape and endeavouring to assess which are particularly central to the wider scientific community [47, 48]. The aims are, again, to increase the visibility of biodata resources and to mobilize research funders to collectively explore how key resources can become financially sustainable over the long term. While beyond the scope of this article, the varied approaches that different resources have taken to fundraising can be understood as a further strategy for overcoming a lack of funding. Licensing, crowdfunding, subscription models, and the privatization of some biocuration have all been suggested as possibilities [49], though many biocurators remain committed to models in which the data that they curate is freely available.

These efforts to increase the visibility of biocuration, professionalize the biocuration community and identify ways of crediting biocuration contributions are extremely important. In different ways, they all seek to allow biocuration to be rewarded within existing research systems—for instance, by enabling biocurators to publish articles or by making biocuration visible in ORCID profiles. In the remainder of this section, however, I want to offer some more speculative proposals for strategies to overcome the challenges biocurators face. These are less oriented to adapting biocuration, as a field of practice, to mainstream funding and evaluation structures (which many scholars have argued are deeply flawed [50]), and more to observing the ways in which the challenges facing biocuration can be understood as representative of wider patterns, and critiqued as such.

One strategy might be to highlight the epistemic contribution that biocuration makes to the biosciences. As noted, one widely held assumption appears to be that curation is essentially ‘clerical’ in nature, oriented to cataloguing existing bioscience knowledge but not to itself shaping knowledge production. It is this view that leads to biocuration’s framing by funders as ‘not research’—as not involving epistemic novelty or innovation. While it is likely that there will be different views from biocurators themselves on the extent to which this is the case, I would argue that the work carried out by biocurators is, in fact, epistemic (involved in knowledge production) in nature in that it renders the biosciences thinkable and usable. In very practical ways, resources shape how future scientific research can be carried out: if, as we have seen, biocuration is fundamental to adding value to bioscience data and to allowing new insights to be derived from it, then even decisions regarding which literature and topics to curate, and which not (for instance), will be central to the directions in which research can go. Indeed, philosophers such as Sabina Leonelli have argued that the development and implementation of bio-ontologies are particularly central to shaping bioscientific knowledge [51, 52]. In discussing bio-ontologies as a form of ‘classificatory theory’, Leonelli suggests that:

Researchers who use bio-ontologies for data retrieval implicitly accept, even if they might not be aware of it, the representation of biological entities and processes contained within bio-ontologies at the moment in which they are consulted. This can have dramatic effects on how data are used in subsequent research … it is because of this crucial role in expressing available knowledge of biological phenomena, and thus guiding and structuring subsequent research, that bio-ontologies are best regarded as a form of theory [52].

In other words, biocuration is hugely significant for how wider research in the biosciences is thought about and can be developed—something that is confirmed by the attentiveness that biocurators give to updating bio-ontologies and to ensuring that older data in resources are revisited, and annotations brought in line with current knowledge. This is vital because ontologies and resources have impacts on work-in-progress science, something that biocurators are, of course, aware of but which is rarely framed as an epistemic contribution. Curation is thus not only about ensuring accessibility to data, but centrally contributes to knowledge production. Perhaps emphasizing this role might help further clarify its research contribution to the biosciences, both to funders and users.

A further approach might be to explore commonalities across different areas of both research-oriented and non-research-oriented data work, and to see if there is scope for collective learning or action. There are striking similarities across these different areas (which range from data annotation on machine learning datasets to data work in healthcare settings), in particular with regard to the devalued and often invisibilized status of this work [53]. Perhaps, then, biocurators might join with others in similar positions in arguing for the need to better support data work and data workers, or make use of concepts that have been mobilized in other areas.

Such concepts include ‘fauxtomation’, which is used to describe what Astra Taylor has called the ‘charade of automation’ [54–56]. There is a wide range of spaces in which users or policymakers are happy to believe that AI or other forms of automation are carrying out tasks which in fact continue to need human involvement, from bots that require humans to (invisibly) step in at critical points to the use of human data annotators or content moderators by companies such as Facebook or Open AI [57]. Such fauxtomation has clear parallels with user and research funders’ frequent assumption that biocuration is a fully automated process, and perhaps similarly speaks both to excessive optimism regarding what technology can achieve (what Mager has termed ‘techno-euphoria’ [58]) and dynamics in which automation tends not to replace humans but to redirect the need for their labour to new sites [56, 59]. In the case of biocuration, fauxtomation occurs as users or funders assume that computable resources have been (or can be) solely constructed through computational methods, thus erasing the work of biocurators from view. Similarly, there are parallels with what has been termed ‘ghost work’—the activities of the data workers who lie behind such apparently automated processes. In their book of the same title, Mary L. Gray and Siddharth Suri define ghost work as the ‘human labour powering many mobile phone apps, websites, and artificial intelligence systems … Left unchecked, the combination of ghost work’s opaque employment practices and the shibboleth of an all-powerful artificial intelligence could render the labour of hundreds of millions of people invisible’ [60]. Those carrying out ghost work—sometimes also termed microwork—might remove offensive content from training data, tag or label images, or check or train AI tools [54, 61, 62]. Such tasks require varying degrees of experience and background knowledge but are ‘ghostly’ in the sense that they are rendered invisible, often being carried out via platforms such as Amazon’s Mechanical Turk and outsourced to sites far away from where the results of such labour are used [63]. Again, we might see parallels with the way in which biocuration allows for remote, distributed work, as well as its invisibility to those who use the data or resources that result from it.

While ghost work is often framed as unskilled, there are other sites in which data work may have clearer parallels with biocuration as a form of expert labour. For instance, data collection, management, curation, and analysis are becoming increasingly central to healthcare [64], with new tasks relating to data collection and management being given to healthcare workers as well as entirely new roles—such as medical scribes—emerging [65]. Just as with biocuration, there have been concerns regarding the ‘proliferation’ of health-related data and a policy imagination that this is straightforwardly usable: as Green *et al*. write, ‘the image of seamless data integration and repurposing keeps flourishing in policy reports’ [66]. And, just as for biocuration, much of the work that is involved in managing this data is rendered invisible, unaccounted for within policy or funding. While data workers within healthcare are perhaps more diverse as a group than biocurators, given that they are spread across a variety of clinical, administrative, research, and repository settings [64, 66], perhaps there is common ground with regard to the challenges that such workers face, and in the expert knowledge that is often required to successfully carry out these forms of data work. As with learning from discussions centred on other forms of data work, one strategy for biocurators may be to explore these parallels and the approaches that are being used to gain recognition for under-valued and invisibilized data labours [61, 67].

In sum, in answering the question ‘What strategies can be used to overcome these challenges?’, I have identified a variety of existing and speculative approaches. Existing approaches include professionalizing and raising the visibility of biocuration and finding means of crediting biocuration activities via authorships and in researcher identifiers. More speculative strategies emphasize the epistemic contributions made to the biosciences by biocuration, and explore parallels and potential learning from other forms of data work.

## Limitations and conclusions

This article has explored contemporary experiences of, and perspectives on, working in biocuration. The aim has been to synthesize research and knowledge regarding the field and, in particular, to discuss the challenges that biocurators face. While I have sought to capture taken-for-granted knowledge about the nature of biocuration, career experiences, and challenges of working in biocuration, a central focus has been existing and potential strategies for overcoming these challenges. Overall, biocurators express a high degree of satisfaction with their work and see it as central to the wider biosciences. Indeed, one way of framing biocuration is as the set of activities that add value to bioscience data, allowing the large amounts of data that are being produced within science to be more fully utilized and insights developed from them. However, the central challenge that biocurators face is the underfunding and under-recognition of this work. While a variety of approaches to meeting this challenge are under development, from gamifying curation and making achievements visible through leaderboards to consolidating the landscape of global biodata resources, it seems likely that broader structural change within research systems will be necessary if biocuration is to be more fully supported and appreciated. Funders must find new mechanisms for sustainably resourcing activities, such as biocuration, that may be understood as providing infrastructure or as dissemination rather than as what is traditionally seen as ‘research’ or ‘innovation’.

While I have attempted to summarize and synthesize knowledge about biocuration, this article should not be understood as comprehensive. In particular, the materials I have drawn on tend to emerge from key organizations and institutions—such as the ISB or the research organizations that host larger groups of biocurators—and from the global North. While this may be representative of the current culture of biocuration, which has been shaped by the history of institutions such as the Swiss Institute of Bioinformatics [68] and by the growth of central resources such as the Gene Ontology or GenBank [1, 16, 69], it is important to note that in practice biocuration looks more heterogeneous than the patterns that I have charted. I have also largely ignored the biocuration work carried out within industry settings: while some interviewees were based in companies rather than in academia, these tended to be those that were closely connected to academic settings and that had many similarities with these. While there are challenges in accessing the kinds of biocuration activities that take place within large multinational corporations (such as pharmaceutical companies), industry settings are a vital aspect of the landscape of biocuration and should be a focus for further research. Similarly, future research could valuably explore global and other forms of diversity in biocuration, as well as building on the arguments presented here to further develop concrete policy proposals for ensuring a better funding environment and a more sustainable future for biocuration.

## Supplementary Material

baaf003_Supp

## Data Availability

The data underlying this article cannot be shared publicly due to ethical and privacy reasons (see discussion in Materials and Methods).
