# Supplementary material for: Working in biocuration: contemporary experiences and perspectives
Source: Database (Oxford). 2025 Feb 12;2025:baaf003. doi: 10.1093/database/baaf003 (PMC11817794; doi:10.1093/database/baaf003)
Supplement: baaf003_Supp [file baaf003_supp.zip › suppl_data/Interview topic guide.docx]

Interview topic guide

Introduction:

- Thank you for your time and willingness to talk to me → confidential and anonymized interview. You can withdraw from the interview - and the research as a whole - at any point.
- Our research: an exploratory interview study that focuses on histories and futures of biocuration. We’re trying to understand both the history of the field as a whole and the trajectories that individuals have taken into it.
- Check if it is okay to record? We will transcribe the interviews but store the audio and text files only on secure GDPR-compliant servers. Interviews will be anonymised at the point of transcription.

Questions:

**Biocuration’s past:**

- How would you describe the history of the field? Where did it emerge from?
  - What were the key dynamics that led to today’s biocuration?
  - Who was involved in the development?
  - Were there particular people, institutions, or databases involved?
  - How was biocuration as a field institutionalized?
  - How did community-building take place?

**Current landscape:**

- What does the current landscape of biocuration look like?
  - Are there any key issues or debates?
  - Does everyone working as a biocurator call themselves that/identify with the biocuration community, and if not, why not?
  - What are typical job titles or descriptions of biocurators?
  - What is the geographical spread of biocuration? Are there any key differences worldwide?

**Individuals’ past:**

- Maybe you could tell me a little bit about how you became a biocurator?
  - What led you into biocuration? (And did you think of it as ‘biocuration’ at that point?)
  - What training did you receive?
  - What seemed to you as rewarding in this career path?
  - What seemed to you as difficult in this career path?
  - How do you fit in with regard to the history of biocuration that you described earlier?

**Future of biocuration:**

- Where do you see biocuration going in the future?
  - What developments are currently taking place that will have effects on biocuration’s future?
  - What training for biocuration is needed in the future?
  - How can the biocuration community be strengthened and fostered?
  - How will automation change the work of biocurators?
- A final, maybe provocative, question: Will we still need biocuration in the future and why?

**Closing:**

- Is there anything important about biocuration’s histories and future that we haven’t discussed so far?
- Who are other important people you think we should speak to?
- Do you have any questions for us?
- Thank you for your time! We would like to keep in touch and will let you know when we have any early results or are giving presentations based on the research.
